# Acceptability of Home-Assessment Post Medical Abortion and Medical Abortion in a Low-Resource Setting in Rajasthan, India. Secondary Outcome Analysis of a Non-Inferiority Randomized Controlled Trial

**DOI:** 10.1371/journal.pone.0133354

**Published:** 2015-09-01

**Authors:** Mandira Paul, Kirti Iyengar, Birgitta Essén, Kristina Gemzell-Danielsson, Sharad D. Iyengar, Johan Bring, Sunita Soni, Marie Klingberg-Allvin

**Affiliations:** 1 Department of Women’s and Children’s health, Uppsala University, Uppsala, Sweden; 2 Department of Women’s and Children’s Health, Division of Obstetrics and Gynecology, Karolinska Institutet/ Karolinska University Hospital, Stockholm, Sweden; 3 Action Research & Training for Health (ARTH), Udaipur, Rajasthan, India; 4 Statisticon, Stockholm, Sweden; 5 School of Education, Health and Social Studies, Dalarna University, Falun, Sweden; NHS lothian and University of Edinburgh, UNITED KINGDOM

## Abstract

**Background:**

Studies evaluating acceptability of simplified follow-up after medical abortion have focused on high-resource or urban settings where telephones, road connections, and modes of transport are available and where women have formal education.

**Objective:**

To investigate women’s acceptability of home-assessment of abortion and whether acceptability of medical abortion differs by in-clinic or home-assessment of abortion outcome in a low-resource setting in India.

**Design:**

Secondary outcome of a randomised, controlled, non-inferiority trial.

**Setting:**

Outpatient primary health care clinics in rural and urban Rajasthan, India.

**Population:**

Women were eligible if they sought abortion with a gestation up to 9 weeks, lived within defined study area and agreed to follow-up. Women were ineligible if they had known contraindications to medical abortion, haemoglobin < 85mg/l and were below 18 years.

**Methods:**

Abortion outcome assessment through routine clinic follow-up by a doctor was compared with home-assessment using a low-sensitivity pregnancy test and a pictorial instruction sheet. A computerized random number generator generated the randomisation sequence (1:1) in blocks of six. Research assistants randomly allocated eligible women who opted for medical abortion (mifepristone and misoprostol), using opaque sealed envelopes. Blinding during outcome assessment was not possible.

**Main Outcome Measures:**

Women’s acceptability of home-assessment was measured as future preference of follow-up. Overall satisfaction, expectations, and comparison with previous abortion experiences were compared between study groups.

**Results:**

731 women were randomized to the clinic follow-up group (n = 353) or home-assessment group (n = 378). 623 (85%) women were successfully followed up, of those 597 (96%) were satisfied and 592 (95%) found the abortion better or as expected, with no difference between study groups. The majority, 355 (57%) women, preferred home-assessment in the event of a future abortion. Significantly more women, 284 (82%), in the home-assessment group preferred home-assessment in the future, as compared with 188 (70%) of women in the clinic follow-up group, who preferred clinic follow-up in the future (p < 0.001).

**Conclusion:**

Home-assessment is highly acceptable among women in low-resource, and rural, settings. The choice to follow-up an early medical abortion according to women’s preference should be offered to foster women’s reproductive autonomy.

**Trial Registration:**

ClinicalTrials.gov NCT01827995

## Introduction

Abortion is legal in India up to 20 weeks of gestation [1], however unsafe abortions are still a major contributor (8–18%) to the maternal mortality ratio (168/ 100 000 live births) [2]. Medical abortion is not yet widely used by the Indian health system although trained doctors, working in public or licensed private health facilities are allowed to provide medical abortion up to 63 days of gestation [3, 4]. The current national medical abortion guidelines mandate three clinical visits carried out by a physician [5], while the World Health Organisation guideline suggests that only one clinical visit is required [6]. Mandatory follow-up visits rarely detect conditions women cannot detect themselves and the timing of follow-up rarely coincides with possible complications after an abortion. Instead, routine follow-up visits result in unnecessary direct and indirect costs for both women and health systems [7]. However, it is clinically important to establish the outcome of the abortion to avoid on-going pregnancies [6, 7]. To increase convenience for women and providers, current literature suggests means of simplifying medical abortion, such as home-use of misoprostol, established to be feasible in both high and low-resource settings [8–11], and alternative methods of follow-up [12–16]. The use of a low-sensitivity pregnancy test (LSPT) at two weeks after a medical abortion is safe and effective in discovering on-going pregnancies [12]. The LSPT can be done at home, followed by a phone call from the clinic, assessing the outcome of the abortion based on the test [12, 17–19]. Results from this randomized control trial (RCT) establish that women’s home-assessment using a LSPT and a pictorial instruction sheet two weeks after medical abortion is as effective as in-clinic assessment by a doctor. Women’s home-assessment had a higher abortion success rate (95%) compared with women in the clinic follow-up group (93%), and thus proves efficacy of simplified medical abortion also in a low-resource and rural setting [20].

Early medical abortion using mifepristone and misoprostol is safe and effective (>95%) [21, 22] and has the potential to enable access to safe abortion services. However, acceptability is an important dimension of access, reflecting the client—provider relationship and more importantly the contextual adaptation of services [23]. Aspects such as confidentiality, decreased numbers of mandatory visits and affordability of services influence acceptability of medical abortion [8, 24, 25]. Additionally, women seeking medical abortion tend to not return to the clinic for their routine follow-up [7, 11]. Studies evaluating acceptability of simplified follow-up after medical abortion have focused on high-resource or urban settings where telephones, road connections, and modes of transport are essentially available and where women have formal education [15, 17, 26]. Hence, it is of great importance to evaluate factors influencing access to safe abortion in a low-resource setting. What is the acceptability of simplified services in a context where women’s autonomy and mobility is limited, educational attainment is low and access to sanitation, electricity, and health care is poor? This study investigates women’s acceptability of home-assessment of abortion and whether acceptability of medical abortion differs by in-clinic or home-assessment of abortion outcome in a low-resource setting in India.

## Materials and Methods

### Trial design

We conducted a randomized controlled non-inferiority trial (RCT) with the secondary outcome to measure acceptability of home-assessment after medical abortion. However, the trial was primarily designed to measure efficacy of home-assessment. The RCT was conducted at six clinics (three rural, three urban) in a low-resource setting in Rajasthan, India. The study protocol and trial is reported according to the CONSORT guidelines for non-inferiority randomised trials [27]. The details of study implementation, setting and context in addition to a thorough explanation of the method are described in the study protocol [28] and the efficacy of the intervention is published elsewhere [20].

### Study setting and participants

The three rural clinics are situated in an interior rural area characterized by limited road connections, high level of poverty, and strong gender disparities including lower educational attainment among women (49% literacy) [29]. Half the population residing in this area belongs to the marginalized social groups, the scheduled castes (SC) and scheduled tribes (ST) [30]. The three urban clinics cater largely to urban women from a broader socio-economic spectrum. All women seeking abortion at the six facilities were screened for eligibility, given that they opted for medical abortion. Women were eligible if they presented with a pregnancy up to nine weeks of gestation as estimated by bimanual pelvic examination; resided in an area where follow-up was possible (within approximately 50 km of the study sites) or had a phone of their own; and agreed to follow-up at two weeks. Women were excluded if they had any contraindications to medical abortion, had a haemoglobin value below 85 mg/l and if they were below18 years of age.

### Intervention and procedure

A research assistant explained the study procedure and obtained written informed consent from eligible women after the administration of mifepristone. Women who consented to participate were randomly allocated to either return to the clinic for follow-up by a doctor (clinic follow-up group), or to assess their abortion outcome at home using an LSPT and a pictorial instruction sheet (home-assessment group). The LSPT used in this study was a DUO-test with a high-sensitivity (5 units/litre) and a low-sensitivity (1000 units/litre) component produced by VEDA Lab (www.vedalab.org). All women could opt for misoprostol administration at home, given that the doctor found it appropriate judging from gestational age, Hb-value and distance from the woman’s house to the clinic. A research assistant contacted women carrying out home-assessment over phone or at home. All women were followed up at 10–15 days after mifepristone administration and women that did not return or could not be contacted (maximum three attempts) within one month after the medical abortion were considered lost to scheduled follow-up and were excluded from analysis.

A doctor or trained midwife assessed women in the clinic follow-up group at the time of follow-up, 10–14 days after mifepristone administration. Abortion outcome was established by the LSPT carried out by a health care provider or bimanual pelvic examination, depending on provider preference and standard practice. Side effects and remaining pregnancy symptoms were evaluated using a standardized questionnaire. All women that returned to the clinic for follow-up were reimbursed for travel expenses. Women in the home-assessment group had been instructed to use the LSPT 10–14 days after mifepristone administration and were subsequently followed up by a research assistant no later than 15 days post mifepristone administration. Phone follow-up was applied where possible, and women with no phone were followed-up by making a discrete home visit, or a visit according to the woman’s preference. Research assistants used a standardized questionnaire to assess abortion outcome by asking the women of their LSPT result, side effects and remaining pregnancy symptoms. If the woman had not done her LSPT, if she had carried out the test too early (within 10 days mifepristone administration) or if she was unsure of the result, the research assistant would ask the woman to carry out the test, provide a new test to repeat the test or ask her to return to the clinic. If it was a phone follow-up, the research assistant would call back later after the woman had carried out the test or ask her to return to the clinic for further assessment. Subsequently, research assistants asked questions regarding unscheduled visits, side effects, abortion experience, and acceptability of the abortion procedure following a standardized questionnaire used for all women.

### Study outcomes and measures

The primary outcome was women’s acceptability of the location of follow-up measured at the time of follow-up and compared between study groups. Acceptability was measured as women’s future preference of follow-up location, overall satisfaction (satisfactory/ not satisfactory); women’s experiences compared to expectations (better/ same/ worse), and women’s comparison with previous abortion experiences when applicable (better/ same/ worse). Moreover, future preference of abortion method (medical/ surgical/ either or) was asked. Additionally, travel time, total time spent in the clinic, number of visits, location of misoprostol, unscheduled visits, adverse events, abortion outcome, socio-demographic and reproductive background of the women and whether they were accompanied to the clinic on day one of the abortion were considered as influential factors and used in the evaluation of factors associated with acceptability. Adverse events were defined as conditions requiring hospitalization, blood transfusion, intravenous fluids or intravenous antibiotics. Secondarily, the feasibility of the use of the LSPT among home-assessment group at follow-up was investigated in comparison with acceptability of home-assessment and the overall acceptability.

### Sample size

The sample size was estimated based on the success rate of medical abortion (95%) [20, 28] to prove the non-inferiority of home-assessment after medical abortion compared with clinic follow-up. The non-inferiority margin (delta) for successful abortion was set to 5% and was based upon perceived clinical significance [18]. With a two-sided significance level α = 0.05, a power of 80%, and an estimated lost to follow-up of 20% the total required sample size of was 716 women. A total of 731 women were recruited between 23 April 2013 and 15 May 2014 and followed up by 15 June 2014. This study measures the acceptability of the intervention and was a secondary outcome of the RCT.

### Randomisation and masking

A computerised random number generator (random allocation software 2.0) generated the randomisation list [31]. Details of randomisation are recorded in the study protocol [28]. Block randomisation with blocks of six was applied to ensure equal distribution over the clinics. The research assistants carried out the randomisation procedure and the group allocation was concealed until the envelopes were opened. Blinding was not possible due to the nature of the intervention. However, the initial clinical assessment by a doctor and mifepristone administration occurred before randomisation. Location of misoprostol administration too, was decided upon before randomization. This decision was made according to the woman’s preference in combination with the doctors’ advice.

### Statistical methods and analysis

All statistical calculations were made using SPSS (version 20) and R (version 3.0.3). Descriptive statistics are presented for all variables. Categorical variables are compared using χ^2^-test or Fischer’s exact test when appropriate. Continuous data was presented as mean (SD). P-value below 0.05 or 95% confidence interval (95% CI) illustrates significant differences. Odds ratios (OR) for the acceptability variables were derived using logistic regression with different explanatory variables. Where OR were significant, adjusted odds ratios (AOR) were derived with multivariate logistic regression.

The study was developed and coordinated by Karolinska Institutet, and Uppsala University, Sweden and Action Research and Training for Health (ARTH), Udaipur, India. The trial was registered at Clinicaltrials.gov: NCT01827995.

## Results

**Fig 1:**
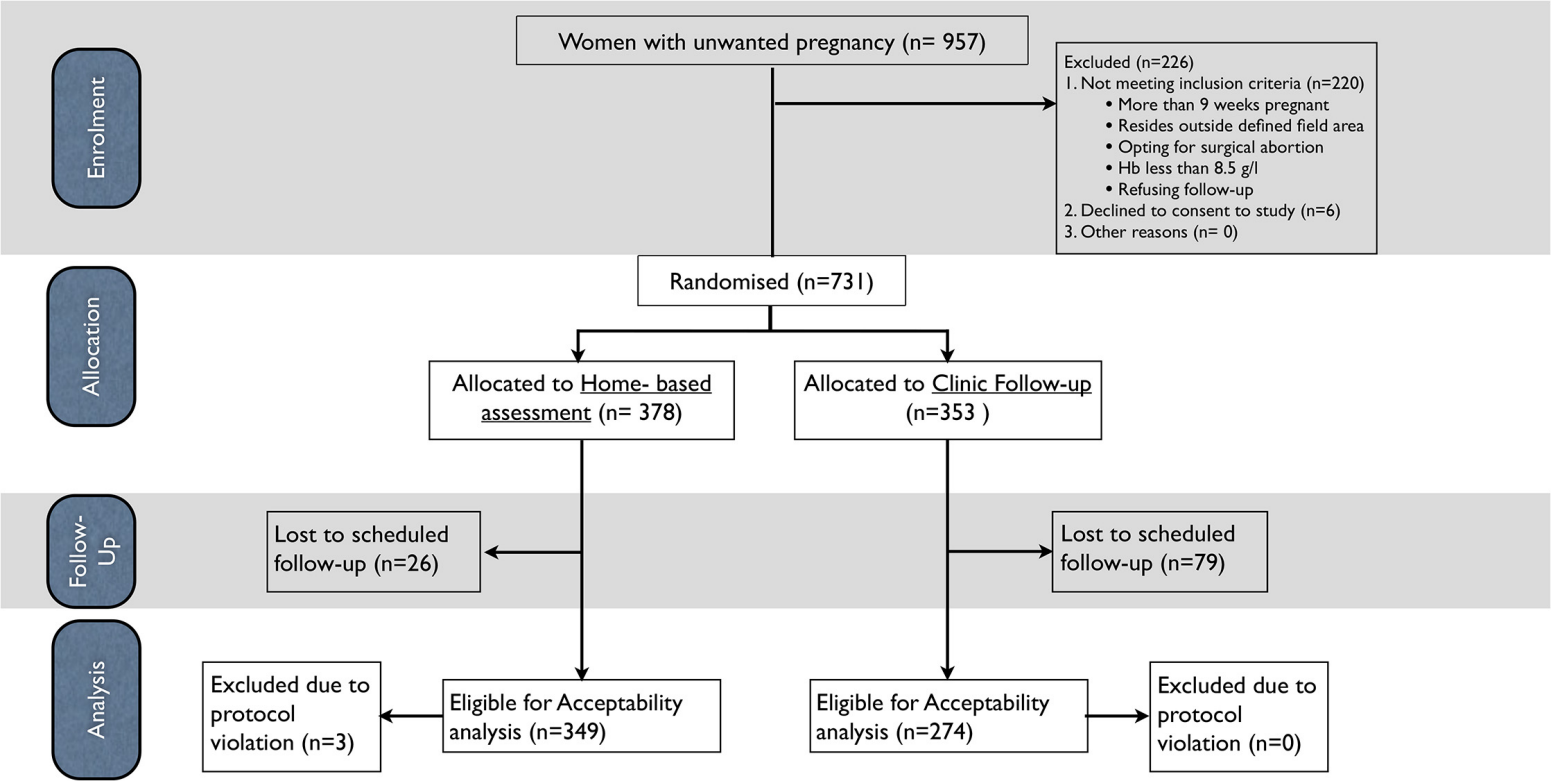
Flow diagram of the RCT. Flow diagram of the study showing enrolment, randomized allocation to home-assessment (n = 378) or clinic follow-up (n = 323) group, two-week follow-up and analysis (n = 623).

A total of 957 women seeking induced abortion were screened for eligibility. Of these, 731 women consented to participate in the study and were randomly assigned to either clinic follow-up group (n = 353) or home-assessment group (n = 378). A total of 626 women were successfully followed-up and the women that complied with the medicine regimen of taking both mifepristone and misoprostol and had a successful scheduled followed-up (n = 623) were analysed for acceptability. The home-assessment group had a better follow-up rate (92%) than the clinic follow-up group (78%). The flow of patients is summarised in a flow diagram (Fig 1).

### Socio-demographic background and reproductive history of participants

Women (n = 623) had a median age of 26, and a median gestational age of six weeks. The majority (71%) resided in rural areas, belonged to the SC/ST social group (55%), and had no formal education (52%). Most women (95.5%) had already given birth and 34% had experienced a previous elective abortion (Table 1). There were no statistical differences between study groups among the successfully followed-up women. The profile of women excluded from acceptability analysis is described in Table 1.

**Table 1:** Characteristics of successfully followed-up women, included for acceptability analysis, stratified by treatment group (n = 623).

	Clinic Follow-up n = 274	Home-assessment n = 349	All Women included n = 623	All Women not included n = 108
**Median age, years (range)**	26 (18–48)	26 (18–46)	26 (18–48)	26 (18–40)
**Residency, n (%)**				
- Urban	92 (33.6)	92 (26.4)	184 (29.5)	15 (13.9)
- Rural	182 (66.4)	257 (73.6)	439 (70.5)	93 (86.1)
**Belong to SC/ST Caste, n (%)**	141 (51.5)	199 (57.0)	340 (54.6)	70 (64.8)
**Level of Education, n (%)**				
- No formal	124 (45.3)	199 (57.0)	323 (51.8)	82 (75.9)
- Primary (1–3 years)	51 (18.6)	49 (14.0)	100 (16.1)	13 (12.0)
- Secondary (4–10 years)	61 (22.3)	57 (16.3)	118 (18.9)	9 (8.3)
- Higher (> 10 years)	38 (13.9)	44 (12.6)	82 (13.2)	4 (3.7)
**Ownership of phone**				
- Woman herself	130 (47.4)	152 (43.6)	282 (45.3)	42 (38.9)
- Husband	100 (36.5)	133 (38.1)	233 (37.4)	36 (33.3)
- No/others	44 (16.1)	64 (18.3)	108 (17.3)	30 (27.8)
**Person accompanying woman to the clinic on day 1**				
-Nobody	94 (34.3)	107 (30.7)	201 (32.3)	42 (38.9)
-Husband	98 (35.8)	117 (33.5)	215 (34.5)	24 (22.2)
-Health Worker^a^	28 (10.2)	51 (14.6)	79 (12.7)	11 (10.2)
-Maternal family member	18 (6.6)	30 (8.6)	48 (7.70)	17 (15.7)
-In-law family member	31 (11.3)	33 (9.5)	64 (10.3)	9 (8.3)
-Neighbour/friend/other	5 (1.8)	12 (3.4)	17 (2.7)	3 (2.8)
**Primigravida, n (%)**	10 (3.6)	18 (5.2)	28 (4.5)	8 (7.4)
**Median gestational age, weeks (range)**	6 (5–9)	6 (5–9)	6 (5–9)	7 (5–9)
- Gestational age in weeks, n (%)				
< 6 weeks	51 (18.6)	58 (16.6)	109 (17.5)	19 (17.6)
6–7	150 (54.7)	196 (56.2)	346 (55.5)	61 (56.5)
> 7 weeks	73 (26.6)	95 (27.2)	168 (27.0)	28 (25.9)
**Prior elective abortion, n (%)**	106 (38.7)	104 (29.8)	210 (33.7)	27 (25.0)
- Medical^b^	79 (28.8)	84 (24.1)	163 (77.6)	19 (70.4)
- Surgical^b^	30 (10.9)	25 (7.2)	55 (26.2)	9 (33.3)
**Home administration of misoprostol, n (%)**	134 (48.9)	156 (44.7)	290 (46.5)	37 (34.3)
**Ever-used modern contraception, n (%)**	111 (40.5)	106 (30.4)	217 (34.8)	25 (23.1)

There were no socio-demographic background differences between the women included in analysis and the women that were lost to follow-up (data of women lost to follow-up is not shown).

^a^ Majority were ASHAs (incentive based community health workers), however NGO workers or auxiliary nurse-midwives occasionally accompanied women as well.

^b^Percentages are calculated for women with prior elective abortions (n = 210)

### Acceptability of simplified follow up

Majority, 355 (57%) women preferred home-assessment in the event of a future abortion. Significantly more women (82% ± 4.0%) in the home-assessment group (n = 349) preferred home-assessment in the future, as compared with 70% (± 5.4%) of women in the clinic follow-up group (n = 274), who preferred clinic follow-up in the future (p < 0.001).

### Satisfaction and abortion experience compared between study groups

Almost all women (96%; 95% CI [93.9–97.2]) were satisfied with their medical abortion and there was no difference in level of satisfaction between the two study groups (Table 2). Most women (95% ± 1.7%) found the procedure better than expected or as expected, while 29 women found the procedure worse than expected. There were no significant differences in expectations between the study groups (Table 2). Educational attainment or urban/rural residency did not affect satisfaction (data not shown). In all socio-demographic and reproductive background variables in Table 1, the proportion of satisfied women ranged between 94–98%. In the event of a future abortion majority of women would opt for medical abortion (81% ± 3.1%), with no difference between study groups (home-assessment: 82% ± 4.1% and clinic FU: 80% ± 5.3%). However, among the 25 women who were not satisfied with their abortion, only half would opt for a medical abortion in the future (data not shown).

**Table 2:** Satisfaction and expectation of the abortion procedure, stratified by treatment group (n = 623).

	Clinic FU n = 274	Home-assessment n = 349	Total
**Overall Experience (p = 0.991)**			
- Satisfactory	262 (96.0)	335 (96.0)	597 (96.0)
- Not Satisfactory	11 (4.0)	14 (4.0)	25 (4.0)
Total	273 (100)	349 (100)	622 (100)
**Experience compared with expectation (p = 0.923)**			
- Same/ Better	260 (95.2)	332 (95.4)	592 (95.3)
- Worse	13 (4.8)	16 (4.6)	29 (4.7)
Total	273 (100)	348 (100)	621 (100)

Chi-square tests were performed on all categorical variables. There are no significant differences. ‘missing values’ were excluded from analysis

Overall, a majority (54% ± 6.9%) of women with previous experiences from elective abortions found the current experience better than before, especially among women who had experienced a previous surgical abortion (81% ± 11%) (Table 3). Almost all women (94% ± 3.1%) with a previous elective abortion reported overall satisfaction, as did women with no previous elective abortion (97% ± 1.7%) (data not shown).

**Table 3:** Women’s experiences of abortion in relation to previous elective abortions, and stratified by treatment group.

	Clinic FU n = 106	Home-assessment n = 104	Total n = 210
**Compared with any previous elective abortion** ^a^ **(p = 0.695)**			
- Better	57 (54.8)	50 (52.6)	107 (53.8)
- Same	32 (30.8)	34 (35.8)	66 (33.2)
- Worse	15 (14.4)	11 (11.6)	26 (13.1)
Total	104 (100)	95 (100)	199 (100)
**Compared with previous medical abortion (p = 0.829)**			
- Better	36 (46.2)	34 (44.2)	70 (45.2)
- Same	30 (38.5)	33 (42.9)	63 (40.6)
- Worse	12 (15.4)	10 (13.0)	22 (14.2)
Total	78 (100)	77 (100)	155 (100)
**Compared with previous surgical abortion (p = 0.848)**			
- Better	23 (79.3)	19 (82.6)	43 (81.1)
- Same	2 (6.9)	2 (8.7)	4 (7.7)
**-** Worse	4 (13.8)	2 (8.7)	6 (11.5)
Total	29 (100)	23 (100)	53 (100)

Chi-square tests were performed on all categorical variables with n>5. Fisher’s test was applied when the expected cell count was <5. There are no significant differences. ‘missing values’ were excluded from analysis. 8 women have had both surgical and medical abortions and are included in both comparisons.

^a^ Only women that reported previous abortion have been included in analysis.

### Feasibility and acceptability in the home-assessment group

Most women (78% ± 4.7%) did their LSPT before follow-up contact, 93% ± 3.0% interpreted the test correctly and 98% found the test easy to do (Table 4). The remaining women (n = 76) did the test at the time of home follow-up or after a reminder over phone.

**Table 4:** Women’s assessment of the LSPT result compared with the final LSPT result as recorded by the research assistant at follow-up (n = 349).

	Women’s and research assistants results assessment of the LSPT result at follow-up^a^		
**Women’s results assessment of the LSPT before follow-up** ^b^	Positive n (%)	Negative n (%)	Not sure n (%)
Positive	9 (69.2)	4 (30.8)	0 (0)
Negative	1 (0.4)	245 (99.6)	0 (0)
Not sure	0 (0)	11 (78.6)	3 (21.4)
Did the test at follow-up	5 (6.6)	70 (92.1)	1 (1.3)
Total	15 (4.3)	330 (94.6)	4 (1.1)

^a^ 21 women carried out a new test at home follow-up due to insecurity of test result or because the test had been done too early (including the false positive). Remaining women did the test by themselves after a reminder of the research assistant.

^b^ As reported by the woman at the time of follow-up. Not all positive test results were repeated, only those that were done too early. Remaining women that reported a positive test result or were unsure of their result were referred to return to the clinic.

A significantly larger proportion (41%) of women with a positive LSPT result or who were unsure of their LSPT-result (n = 27) preferred clinic or doctor’s advice for follow-up in the event of a future abortion, compared with 14% of women with a negative LSPT result (n = 245) (p<0.000). Future preference of home-assessment was not affected by whether women had carried out the LSPT by themselves or not. However, the odds of preferring future clinic follow-up were slightly higher among women who did not carry out their LSPT by themselves (OR 1.7, 95% CI [1.0–3.2]) (S1 Table). Additionally, women who did not use the pictorial instruction sheet were less likely to prefer home-assessment in the future (AOR 6.1, 95% CI [2.4–15.4]) (S1 Table). Odds ratios were adjusted for; advised return to clinic, thinking the abortion is complete, severe stomach pain, use of pictorial instruction sheet, LSPT result, and abortion outcome. Future preference for home-assessment was not affected by residency (urban 89% ± 6.5% and rural 79% ± 5.0%) or education (Formal 87% ±5.4% and None 77% ± 5.9%). However, women residing in rural areas were significantly more likely to answer “doctors’ advice” in terms of future preference (5.9% ± 2.89%) compared with women residing in urban areas where none answered “doctor’s advise” (data not shown).

Significantly more women (98%) who did the LSPT according to protocol, before the contact with the research assistant, were satisfied with their abortion compared with women who did their LSPT after or during the follow-up contact (88%) (p < 0.000). A higher proportion of women who did their LSPT before follow-up contact thought their abortion was complete (80%) and were less likely to report abortion-related symptoms at follow-up (82%). Correspondingly, believing the abortion was complete at the time of follow-up was significantly associated with overall satisfaction (p = 0.007) while being advised to return to the clinic by the research assistant had a negative impact on satisfaction (p < 0.001) (data not shown).

### Factors influencing overall acceptability of medical abortion

Number of previous births, gestational age and previous elective abortions had no influence on the overall satisfaction (data not shown). However, women were more likely to be dissatisfied if they had an unsuccessful abortion (AOR 18, 95% CI 5.7–57) (S2 Table). Of the women that were not satisfied (n = 25), half had an unsuccessful abortion and needed an additional intervention (manual vacuum aspiration, repeat medical abortion, or additional misoprostol), and one woman chose to continue her pregnancy (Table 5). Moreover, women were less likely to be satisfied with the abortion if they experienced side-effects such as bleeding, vomiting, diarrhoea and severe stomach pain during misoprostol administration on day three (AOR 2.9, 95% CI 1.0–8.3), if women had contacted the clinic due to side-effects or signs of incomplete abortion before follow-up (AOR 4.7, 95% CI 1.7–13), or if women reported any continuing abortion-related symptoms at follow-up (AOR 4.7, 95% CI 1.7–13). Odds ratios were adjusted for; time spent travelling, interim visits, side-effects from misoprostol, additional contact due to complications, additional surgical or medical intervention, reported symptoms present at follow-up, and outcome of abortion (S2 Table).

**Table 5:** Abortion outcome of successfully followed-up women (n = 623) compared with level of satisfaction and expectation of abortion.

	Complete	On-going	Incomplete	Total	
**Overall Experience**					
- Satisfactory	576 (96.5)	2 (0.3)	19 (3.2)	597 (100)	p < 0.001
- Not satisfactory	12 (48.0)	4 (16.0)	9 (36.0)	25 (100)	
Total	588 (94.7)	6 (1.0)	28 (4.5)	622 (100)	
**Compared with expectation**					
- Better	332 (96.2)	2 (0.6)	11 (3.2)	345 (100)	p < 0.001
- Same	241 (97.6)	0 (0.0)	6 (2.4)	247 (100)	
- Worse	14 (48.3)	4 (13.8)	11 (37.9)	29 (100)	
Total	587 (94.7)	5 (0.8)	28 (4.5)	621 (100)	

Chi-square tests were performed on all categorical variables. Fisher’s exact test was performed where expected count was <5. ‘missing values’ were excluded from analysis. All women that had an on-going or incomplete abortion received additional surgical or medical intervention for completion of their abortion.

Increased travel time was associated with not being satisfied (AOR 1.7, 95% CI 1.0–2.8) (S2 Table) and women in the clinic follow-up group spent more time travelling and more time in the clinic compared with the home-assessment group (data not shown).

## Discussion

### Main findings

This study confirms acceptability of medical abortion using a simplified follow-up regime and women’s future preference of home-assessment of abortion outcome. Medical abortion is highly accepted among women in many settings [21, 32, 33]. In addition to previous studies suggesting feasibility of medical abortion in low-resource urban settings [8, 11], our results indicate acceptability of medical abortion with home-assessment after early medical abortion as a follow-up alternative and suggest its appropriateness for scaling up in low-resource, rural settings. Importantly, this study highlights that satisfaction is not determined by educational attainment or lack of resources, enhancing the scope of implementing simplified medical abortion in settings where women have low educational attainment, and family and health system resources are scarce. In line with previous studies [11, 34], our study confirms that satisfaction is primarily affected by the abortion outcome as well as the presence of side effects or abortion-related symptoms rather than means of follow-up.

Simplifying medical abortion in terms of task sharing to mid-level providers’ [35–37], home-use of misoprostol [8, 9] and simplified follow-up [12–14] is effective, safe and accepted in high-resource or urban settings. This study confirms the acceptability and feasibility of simplified follow-up in a low-resource, rural setting, in which the majority of women are illiterate, infrastructure is poor and mobile phone possession among women is rare. Showing acceptability in such a setting suggests acceptability to be generalizable to other low-resource settings.

The use of a pictorial instruction sheet or a checklist has in recent studies of simplified follow-up been regarded as unnecessary or resulting in unnecessary referrals [15, 26]. Findings from our study indicate that women who used the pictorial instruction sheet were more likely to prefer home-assessment in the future. Keeping in mind that, women who used the instruction sheet may have been more informed or motivated to carry out the home-assessment and were subsequently more likely to be satisfied. We used the pictorial instruction sheet as both an instruction of how to use the LSPT and a checklist of danger signs in relation to abortion [28].

For successful implementation of an intervention in a low-resource and complex setting, it is important to understand how clients internalize the intervention [38, 39]. Women in the home-assessment group reported a good overall abortion experience and majority of women correctly carried out and assessed their result of the LSPT. To believe that the abortion was complete, according to the woman’s self-assessment based on the information and tools given to her on day one, and having used the LSPT before the research assistant came for follow-up, were both associated with satisfaction. These findings indicate the value to women of enabling home-assessment as an alternative to clinic follow-up, and further strengthened by the fact that a positive LSPT did not result in the future preference of clinic follow-up. This highlights the feasibility of increasing women’s autonomy in abortion care and its positive effect on acceptability of abortion services. Moreover, it sheds light on the importance of acknowledging that women’s preferences vary, and optimal abortion care must give women a choice of how to assess their abortion outcome.

There was a greater loss to follow-up in the clinic follow-up group, especially among rural residents. This could partly be explained by the study area’s poor road and transport infrastructure and women’s lack of autonomy in their family setting. It is common that women do not return for follow-up after a medical abortion [7, 11, 13] however, the follow-up is of great importance to avoid ongoing pregnancies. This loss to follow-up indicates an implicit need for alternative means of follow-up where women’s choice to assess their abortion outcome at home is a feasible option. Given that women are provided with sufficient counseling of how to carry out the LSPT and are encouraged to seek care if needed. It is known that women seek care through unscheduled visits if they experience side effects or signs of incomplete abortion, rather than waiting for their scheduled follow-up [13]. A study from Nepal highlights the importance of the provision of sufficient information with regard to medical abortion to improve acceptability [40]. Qualitative research to explore women’s and providers’ experiences could contribute to a deeper understanding of acceptability of home-assessment.

A limitation of the study was that women, lost to scheduled follow-up, could not be assessed for acceptability and this may have an effect on the proportion of satisfied women in the clinic follow-up group. However, the high satisfaction in our study corresponds with the high satisfaction reported previously [21, 32, 33]. Moreover, women that returned for clinic follow-up were given a travel reimbursement, to decrease loss to follow-up [28], this may have enhanced satisfaction reported by women in the clinic follow-up group. It is also important to consider that questions regarding acceptability were asked in the clinics for the clinic follow-up group. This may have biased women in their responses of satisfaction, compared with women that were followed-up at home or over phone. Due to the small number of dissatisfied women it is difficult to draw conclusions on characteristics of women that reported dissatisfaction in the different groups.

Considering the lack of access to safe abortion and the large number of unsafe abortions in India [41, 42], simplifying medical abortion through increasing women’s autonomy in abortion care can enforce women’s reproductive rights and enable access to safe, acceptable abortion services. Our study is unique in its contribution of data from a low-resource and rural setting and combined with the adaptation phase [28] and the proven efficacy of home-assessment [20] this study adds important evidence for implementation and scale-up of medical abortion in rural, low-resource settings.

### Interpretation

Early medical abortion with home-assessment using a LSPT and a pictorial instruction sheet at two weeks is acceptable and feasible in a low-resource setting and should be an available choice to women in primary health care facilities in low- and high- resource settings.

## Conclusion

Medical abortion with home-assessment of abortion outcome is highly accepted among Indian women in a low-resource setting where educational attainment is low. Simplified follow-up using a low-sensitivity pregnancy test and a pictorial instruction sheet should be introduced as an alternative for women seeking abortion in both high- and low-resource settings. Simplified follow-up after medical abortion can increase acceptability and accommodate women in need of abortion services. In addition, it may increase women’s autonomy in abortion care and hence ensure women’s reproductive rights through access to safe and acceptable abortion services.

## Supporting Information

S1 CONSORT Checklist CONSORT checklist. (DOC)

S1 Protocol Study protocol. (DOC)

S1 Table Factors among home-assessment group influencing the future preference of follow-up in the event an abortion (n = 349). The table reflects factors associated with future preference of clinic FU or according to doctor’s advice among women in the home-assessment group.
(DOC)

S2 Table Characteristics of women and abortion experience in relation to satisfaction with the abortion procedure. Results are presented as the Odds Ratio (OR) or adjusted OR of being dissatisfied.
(DOCX)
