# Supplementary material for: Acceptability of Home-Assessment Post Medical Abortion and Medical Abortion in a Low-Resource Setting in Rajasthan, India. Secondary Outcome Analysis of a Non-Inferiority Randomized Controlled Trial
Source: PLoS One. 2015 Sep 1;10(9):e0133354. doi: 10.1371/journal.pone.0133354 (PMC4556554; doi:10.1371/journal.pone.0133354)
Supplement: S1 Protocol — (DOC) [file pone.0133354.s002.doc]

**Study protocol 130327**

**Management of medical abortion using simplified follow up. A two-armed randomized controlled trial at district level in Rajasthan, India.**

It is estimated that around 50 000 women die every year due to consequences of unsafe abortion (Singh et al, 2009). A majority of these deaths occur in low-income countries where access to safe abortion care is limited. Unplanned pregnancy and unsafe abortion thus place a huge burden on scarce medical resources. Any improvement in Comprehensive Abortion Care (CAC) would mean important improvements for the concerned women and their families (Singh, 2006) as well as for the societies at large. Recommended improvements concern increased access to the safest and most cost-effective methods such as medical abortion provided at primary level facilities (Singh et al, 2009). A Cochrane Review concludes that most randomised controlled trials within the field of medical abortion are conducted in high-income settings with good access to emergency health care services (Kulier et al, 2004). A simplified regime for medical abortion, which could be used at primary level facilities, would contribute to the reduction of maternal mortality and morbidity related to unsafe abortions globally. However, there is a knowledge gap in order to determine if a simplified follow up of medical abortion is equally effective as the standard procedure in a low-income setting. The results will provide evidence-based information to be used in revising training and service delivery guidelines’ in order to reduce maternal mortality and morbidity in both low and high-income setting.

**Survey of the field**

In collaboration with WHO, *medical abortion* has been developed into a safe and effective method for induced abortion globally (WHO, 2000, WHO, 2001, Fiala et al, 2004, WHO, 2010). Medical abortion using a combination of *mifepristone* and the prostaglandin E1 analogue *misoprostol* has been suggested as an effective tool in conducting first trimester abortions (Weeks, 2001, von Hertzen et al, 2003, Kulier et al, 2004 Weeks et al, 2005, Gemzell-Danielsson et al, 2007, Tang et al, 2007, Blum et al, 2007,). International studies have compared and found no significant differences in effectiveness between medical abortion and the conventional surgical intervention the Manual Vacuum Aspiration (MVA) for treatment of first-trimester abortion (Say et al, 2002, Singh, 2006, Fetters et al, 2008). Medical abortion with the combined regimen of mifepristone followed by a prostaglandin analogue was first developed by the research group at the WHO-collaborating centre at the department of Obstetrics and Gynaecology, Karolinska University Hospital, Sweden (WHO, 2000, WHO, 2001, Fiala et al, 2004). Following previous studies, women now have the option of a simplified procedure of medical abortion with home self administration of misoprostol, also called home based abortion, which has had a major impact in Sweden (Kopp Kallner et al, 2010) leading to increased access and reduced waiting time. For women with an unwanted pregnancy who have decided on an abortion it is of major importance to avoid unnecessary waiting times (Tang et al, 2007). Another possibility to reduce waiting time would be to allow midwives (midlevel providers) to be more involved with care of healthy women undergoing medical abortion. Recently the Board of Health and Welfare in Sweden stressed the need to evaluate the involvement of midwives in medical abortion. In Nepal medical abortion provided by midlevel providers has been shown to be as safe and accepted as abortion care provided by physicians (Warriner, 2011).

A review shows that bleeding, pain, uncertain length of treatment and the number of visits required to the clinic has a major influence on the acceptability of medical abortion among women (Clark, 2007).[[1]](#endnote-2) Today a follow up visit is included in the protocol for medical abortion. However, many women do not return for the follow up visit or refrain from choosing medical abortion due to the requirement for such a visit. A systematic review assessed the evidence regarding follow up after medical and surgical abortion (Grossman, 2010). No direct evidence for Follow up versus no Follow up was available. Post abortal complications such as heavy bleeding, infection, ectopic- or incomplete abortion prompt women, especially if adequately counselled, to seek care outside of a routine follow up visit. Thus the main reason for the routine follow up is to detect an on-going pregnancy and to provide contraceptive counselling. Most clinics already provide contraceptive counselling at the time of the first visit in order to make it possible for the woman to start postabortal contraception without delay.

The review by Grossman et al., focused on the accuracy of follow up protocols to diagnose an on-going pregnancy following medical abortion. Based on analyse of nine studies whereof only one was a RCT it was concluded that women’s self-assessment has varying sensitivity to diagnose on-going pregnancy. Serum hCG testing was found to be an accurate modality to detect ongoing pregnancy but it added both visits and costs of the medical abortion. Therefore, urine pregnancy test (u-hCG) later than one week after the abortion using a low sensitivity, semi quantitative test, may offer an acceptable and safe alternative to existing protocols. Follow up using Urine low sensitivity test has since many years become the standard practice in Sweden. Any improvement in the treatment of abortion has a great impact on women’s reproductive health, in developing as well as in developed countries. Easy access to a simplified regimen of medical abortion and involvement of midlevel providers can contribute to achieve this goal, if high efficacy and safety can be guaranteed.

*The Indian context*

The United Nations Millennium Development Goal (MDG5) number 5 is to decrease maternal mortality ratio by 75% from 1990 to 2015. The maternal mortality ratio in India has declined from 570 (1990) to 230 per 100.000 live births in 2008. The estimated MMR for India is 230, ranging between 150-350 /100,000 births (Center for reproductive rights, 2008, WHO, 2009). India alone is responsible for 20% of maternal deaths worldwide (Population Action International, 2007, Dileep et al, 2008). It is estimated that 17 % of the maternal deaths in India were attributed to unsafe abortions (Iyengar et al, 2002) a proportion considered high in international comparison (Center for reproductive rights, 2008, WHO, 2009).

Already in 1971, India passed the Medical Termination of Pregnancy Act. Despite this, morbidity and mortality from unsafe abortion are still serious public health problems. Safe and legal abortion services remain limited, especially to women in rural areas (Center for reproductive rights, 2008). To achieve the MDG 5, the government of India has identified increasing access to safe abortion services as a strategy to reduce maternal mortality and morbidity under the National Rural Health Mission (Garg, S & Nath,A, 2008). India alone is responsible for 20% of maternal deaths worldwide (Mavalankar, 2008) and unsafe abortion contributes to 8% of all maternal deaths in India (Sample Registration System, 2006). The maternal mortality due to unsafe abortion in India remains high and indicates an urgent need to simplify procedures in order to reduce maternal mortality and morbidity.

According to the National Rural Health Mission, in India, Primary Health Centers (PHC) should provide abortion care service but absence of trained providers and lack of equipment act as barriers. Medical abortions using mifepristone and misoprostol up to 63 days of pregnancy were approved by the Drug Controller of India 2002. Recent study from rural area of India showed that provision of medical abortion by medical doctors is feasible and acceptable at health care facilities that do not offer surgical abortion service (Mundle et al, 2007). However, a recent survey among qualified abortion providers showed that only 3 % currently provided medical abortion and 23 % intended to do so (Andreea et al, 2008). The public sector in India has been slow in implementing medical abortion and it is suggested that a simplified procedure would increase acceptability among both providers and women (Bracken, 2010). However, there is need to provide clinical evidence for a change in the national training and service delivery guidelines’ in India.

*Definitions*

*Standard regime (control group):* Standard procedure medical abortion (Ministry of Health and Family Welfare, 2010) includes three clinical visits between 1-14 days from first visit. First visit is used to asses’ women’s eligibility for medical abortion and supervised administration of mifepristone. At second visit, misoprostol is administered at the clinic. Third visit is between 10-14 days after administration of mifepristone and aims to determine if the induced abortion is complete and to identify complications.

*Simplified follow up (intervention group):* Simplified procedure includes only one or two visits for mifepristone and misoprostol as per clinic’s protocols. They do not routinely return for a follow up visit on day 10-14, instead they are given instructions on how to use a checklist and pregnancy test and they return for follow up only if they have any health problem or screen positively.

**Design and method**

**Hypothesis:** Self-assessment of the complete abortion using a semi-quantitative u-hCG test will be as effective and safe as routine follow-up among women in rural Rajasthan.

**Objectives:**

(1) To assess the safety and efficacy of simplified follow up using a semi-quantitative u-hCG test as compared to the standard regime among women in rural Rajasthan.

(2) To evaluate women’s experiences and acceptability of the simplified follow up using a semi-quantitative u-hCG test among women in rural Rajasthan.

(3) To evaluate the safety and acceptability of self-administration of misoprostol among women in rural Rajasthan.

(4) To compare women’s contraceptive usage post abortion between the groups with simplified follow up and the standard regime.

*Setting:* A total of six facilities will be included in the study. An estimated 800 women receive induced abortion services in these clinics and about 600 of them choose to use medical abortion. The facilities to be included in the study are 4 clinics of a non government organization, Action Research & Training for Health (ARTH), Udaipur and two private clinics located in Udaipur city, India.

*Participants:* All women with a gestational age < 63 days, based on LMP as reported by the women, opting for medical abortion at the study sites will be invited to participate in the study. All participants will be given oral and written information in order to make an informed consent.

*Inclusion criteria:* Women presenting with a positive urine test and uterine size equivalent to or up to 9 weeks of gestation opting for medical abortion and residing in an area where follow-up is feasible. Woman agrees for a follow up contact at 10-14 days.

*Exclusion criteria:* women with contraindications to medical abortions, haemoglobin level less than 85 (Hb <85) and age less than 18 years.

*Data collection:* Abortion providers involved in abortion care at the different study sites will undergo a standardized training programme conducted by ARTH. Eligible women who consent to participation will be randomized to either;

Group A. Standard regime of follow up (control group). Mifepristone at outpatient clinic followed by the administration of misoprostol 24-48 hours later (in the clinic or at home). Follow-up at the clinic on day 10-14 by qualified practitioner to evaluate success of procedure using the clinic’s standard routine.

Group B. Simplified followup (Intervention group) Mifepristone at outpatient clinic, followed by the administration of misoprostol 24-48 hours later (in the clinic or at home). The women will not return to the clinic for routine follow up visit, but are given instructions on how to use a checklist and a low sensitivity pregnancy test around day 10-14. They are asked only to return if they have any health problems or screen positively. The low sensitivity urinary-hCG (with HCG of 1000 IU/ml) test will be used. Women will be provided the pregnancy test free of cost along with a pictorial checklist. A research assistant or a nurse will explain in detail how to use the pregnancy test as well as the checklist, and provide phone numbers in case of any questions or doubts. Follow-up interviews will be conducted through home visits or phone (if woman has one) during the next 1-2 days to screen for ongoing pregnancies and assess acceptability the self-assessment.

The randomization will be conducted in blocks of 6 and will vary randomly. A computer random number generator will be used to generate a list of codes from 1 to 1200 and each code is linked to one of the two groups – A and B. The list will be used while sequentially numbered, opaque, sealed envelopes will be prepared by the research team. Each envelope contains a study protocol for the individual women. At the time of allocation, the research assistant at the clinic will pick the envelope with the lowest number, write the participant’s name and personal registration number on it, and then open it. Process evaluation will be conducted by intermittent check-ups in order to assure that the intervention procedures are performed correctly and that they follow the protocol. The check-ups will be made by the coordinator and included both control of the completed protocols and repetition and education of the abortion providers involved in the study.

Components of clinical assessment are; (i) History taking (socio-demographic information, obstetric history, contraceptive history (ii) Physical examination include general examination (pulse, blood pressure and temperature); (iii) Pelvic examination (External genitalia, speculum examination, Bimanual examination, (iv) Screening for contraindication of medical abortion (v) Laboratory investigations (pregnancy status using urinary hCG and haemoglobin) .

*Outcome:* The outcome measures will be the same for all subjects to establish the efficacy of the intervention and the reference treatment. Established classification of efficacy for medical abortion will be followed to determine the outcome of the abortion procedure (Kopp Kallner et al. 2010). The *main outcome* is; efficacy of self-assessment, measured as the rate of complete abortion, relative to routine follow up. The percentage of women requiring surgical intervention, extra visits and administration of additional misoprostol will be used to monitor incomplete abortion.

*Secondary outcomes* are; (i) acceptability of self-assessment (ii) unscheduled visits for various adverse events/complications/continued pregnancies (iii) side effects of medical abortion including infections, bleeding, pain (iv) contraceptive uptake at 2 weeks after abortion (v) time consumption, (v) women’s acceptability of home-use of misoprostol .

**Follow up:**

The follow up of both groups will be conducted between 10-14 days. The control group will attend the clinic for follow up and the intervention group will either be followed up by telephone or by home visit. The *assessment to measure* the *main outcomes* are: In self assessment group (intervention group), (i) a series of questions to assess side effects / adverse events (ii) women’s self assessment of pregnancy status (low sensitivity U-hCG and symptoms); In reference treatment (control group), (i) a series of questions to assess side effects / adverse events (ii) Physical examination (pulse, blood pressure and temperature); (iii) Pelvic examination if needed (External genitalia, speculum examination, Bimanual examination, (iv) Pregnancy status using low sensitivity urinary hCG

*Measurements of secondary outcomes are*: Standardized questionnaires will be used to collect information on time spend on travelling and on clinical visits, contraceptive use pre and post abortion, women’s acceptability of the self-assessment and the home use of misoprostol and the abortion method (Fiala et al. 2004). Assessment to measure main and secondary outcomes will be collected at follow up visit after 10-14 days for the two groups.

**Sample size estimate**:

A total of 596 women have been calculated to be needed to reach statistical significance (α=0.05 at a power of 80%) and proof the non-inferiority of the intervention. This calculation assumes a percentage of women requiring surgical treatment after medical abortion of 5% with routine follow-up (Von Hertzen H et al, 2010).

It is estimated that about 20% of women will be lost to follow-up, resulting in a total of about 716 women that will have to be recruited.

The formula for calculating sample size based on fixed power is as follows

Power = 1 - Phi(Z-Delta/(Var^(1/2))) + Phi(-Z-Delta/(Var^(1/2)))

Where:

- ”Phi” represents standard normal CDF and
- Z = Standard normal quantile at the desired level of significance (in this study Z=1.96, for 5% level of significance)
- Delta = Margin of error (in this study Delta=0.05 )
- Var = p1*(1-p1)/(n/2) + p2*(1-p2)/(n/2),  in your case p1 = p2 = 0.95 (because, you mentioned, success rate of medical abortion is 95%)
- n = required sample size (i.e. n/2 from each of two comparison groups)

For a fixed power, 0.8, this formula gives an approximate sample of size 596 (i.e. 298 in each group).

For 20% expected non-response, we may increase the sample size as 596*1.2=716.

The non- inferiority of self-assessment of complete abortion following medical abortion as compared to the routine follow-up will be established by using a non-inferiority margin (delta) of 5% for the difference in percentage of women requiring surgical abortion from the two groups.

The sample size estimation is based proofing non-inferiority of the primary outcome; the secondary outcomes will be addressed in addition to the primary outcome, however using the same sample size.

**Statistical analyses:** All analyses will be by Intention to treat (ITT). The non-inferiority hypothesis will be tested at a significance level of 5% (α=0,05). Categorical outcomes will be presented using descriptive statistics and compared using Xai-square test and numerical variables will be assessed for significance using student’s t-test. Intergroup comparison will be made between the reference treatment group and intervention group. Differences between groups will be analysed using relative risks (95% CI) and logistic regression for the relative differences. P-values equal to or lower than 0.05 will be considered statistically significant. Acceptability measures will be analysed descriptively, using numbers and percentages of women in the different categories.

**References**

Andreea A, Creanga, Priyadarshini Roy, Amy O, Tsui. Characteristics of abortion service providers in two northern Indian states. *Contraception* 78; 500-506, 2008.

Berer M. [Provision of abortion by mid-level providers: international policy, practice and perspectives.](http://www.ncbi.nlm.nih.gov.www.bibproxy.du.se/pubmed/19197405?itool=EntrezSystem2.PEntrez.Pubmed.Pubmed_ResultsPanel.Pubmed_RVDocSum&ordinalpos=1) *Bull World Health Organ*. 2009 Jan;87 (1):58-63.

Blum J, Winikoff B, Gemzell-Danielsson K, Ho PC, Schiavon R, Weeks A: Treatment of incomplete abortion and miscarriage with misoprostol. *Int J Gynaecol Obstet* 2007, 99 Suppl 2:S186-189.

Bracken Hillary. Home administration of misoprostol for early medical abortion in India. *Int J Gynaecol Obstet* 2010, 108:228-232.

Center for reproductive rights, 2008. Maternal mortality in India. New York, USA.

Clark, WH, Gold M, Grossman D, Winikoff B. 2007. Can mifepristone medical abortion be simplified? A review of the evidence and questions for future. Contraception 75; 245-250.

Dileep Mavalankar, Kranti Voraa & M Prakasamma. Achieving Millenium Development Goal 5: Is India Serious? *Bulletin of the World Health Organization* 2008, 86(4):243.

Fetters T, Tesfaye S, Clark KA: An assessment of postabortion care in three regions in Ethiopia, 2000 to 2004. *Int J Gynaecol Obstet* 2008, 101(1):100-106.

Fiala C, Winikoff B, Helstrom L, Hellborg M, Gemzell-Danielsson K. Acceptability of home-use of misoprostol in medical abortion. *Contraception* 2004; 70:387-392.

Garg S, Nath A. Current status of national rural health mission. Indian J Community Med, 2008 Jul 29; 32:171-2.

Gemzell-Danielsson K, Ho PC, Gomez Ponce de Leon R, Weeks A, Winikoff B: Misoprostol to treat missed abortion in the first trimester. *Int J Gynaecol Obstet* 2007, 99 Suppl 2:S182-185.

Grossman D, Grindlay K. Alternatives to ultrasound for follow-up after medication abortion: a systematic review. Contraception 2010; *In press.*

Iyengar K, Iyengar SD. Elective abortion as a primary health service in rural India: experience with manual vacuum aspiration. *Reprod Health Matters*. 2002;10:54-63.

Kopp Kallner, C. Fiala , O. Stephansson and K. Gemzell-Danielsson. Home self-administration of vaginal misoprostol for medical abortion at 50–63 days compared with gestation of below 50 days. In press Hum Reprod 2010.

Kruk ME, Pereira C, Vaz F, Bergstrom S, Galea S: Economic evaluation of surgically trained assistant medical officers in performing major obstetric surgery in Mozambique. *Bjog* 2007, 114(10):1253-1260.

Kulier R, Kapp N, Gülmezoglu AM, Hofmeyr GJ, Cheng L, Campana A. Medical methods for first trimester abortion. *Cochrane Database of Systematic Reviews* 2004, Issue 1. Art. No.: CD002855. DOI: 10.1002/14651858.CD002855.pub3.

Machin D, Campbell M, Fayers P, Pinol A. Sample size tables for clinical studies. Oxford: Blackwell Science, 1997.

Medical termination of pregnancy Act (MTP Act), Act No. 34 of 1971. Commercial Law Publishers, India, Delhi. Revised version, 2010.

Ministry of Health and Family Welfare, Government of India; Comprehensive Abortion Care, Training and Service Delivery Guidelines. 2010.

Mundle S, Elul B, Anand A, Kalyanwala S, Ughade S. Increasing access to safe abortion services in rural India: experiences with medical abortion in a primary health centre. *Contraception* 76; 66-70, 2007.

Patel L, Bennet T.A, Tucker Halpern C, Johnston H. B, Suchindran C. M. support for provision of early medical abortion by mid-level providers in Bihar and Jharkand, India. *Reproductive Health Matters.* 2009;17:70-79.

Population Action International. A Measure of Survival - Calculating Women's Sexual and Reproductive Risk. 2007.

Say L, Brahmi D, Kulier R, Campana A, Gülmezoglu AM. Medical versus surgical methods for first trimester termination of pregnancy. *Cochrane Database of Systematic Reviews* 2002, Issue 4. Art. No.: CD003037. DOI: 10.1002/14651858.CD003037.pub2.

Singh S: Hospital admissions resulting from unsafe abortion: estimates from 13 developing countries. *Lancet* 2006, 368(9550):1887-1892.

Singh S et al., Abortion worldwide: A decade of Uneven Progress. New York: Guttmacher Institute, 2009.

Tang OS, Gemzell-Danielsson K, Ho PC: Misoprostol: pharmacokinetic profiles, effects on the uterus and side-effects. *Int J Gynaecol Obstet* 2007, 99 Suppl 2:S160-167.

Warriner IK, Meirik O, Hoffman M, Morroni C, Harries J, My Huong NT, Vy ND, Seuc AH: Rates of complication in first-trimester manual vacuum aspiration abortion done by doctors and mid-level providers in South Africa and Vietnam: a randomised controlled equivalence trial. *Lancet* 2006, 368(9551):1965-1972.

Warriner IK, Wang D, My Huong, Thapa K, Tamang A, Shah I, Baird DT, Meirik O; Can midlevel health-care providersadminister early medical abortion as safely and effectively as doctors? A randomised controlled equivalence trial in Nepal. Lancet 2011; 377:1155-61

Weeks A: Clarifying the role of misoprostol in obstetrics. *Afr Health Sci* 2001, 1(2):52-54.

Weeks A, Alia G, Blum J, Winikoff B, Ekwaru P, Durocher J, Mirembe F: A randomized trial of misoprostol compared with manual vacuum aspiration for incomplete abortion. *Obstet Gynecol* 2005, 106(3):540-547.

WHO. Task Force on Post-ovulatory Methods of Fertility Regulation. Comparison of two doses of mifepristone in combination with misoprostol for early medical abortion: a randomised trial. *British Journal of Obstetrics & Gynaecoloy.* 2000,107:524-530*.*

WHO. Task Force on Post-ovulatory Methods of Fertility Regulation. Medical abortion at 57 to 63 days` gestation with a lower dose of mifepristone and gemeprost. A randomized controlled trial. *Acta Obstetricia et Gynecologica Scandinavica.* 2001, 80:447-451.

WHO. The research group on Post-ovulatory Methods of Fertility Regulation. Misoprostol dose and route after mifepristone for early medical abortion: a randomised controlled non inferiority trial. *BJOG.* 2010,1186-1196:524-530*.*

WHO, 2003. Safe abortion: Technical and policy guidance for health systems. World Health Organization, Geneva.

WHO, 2004. Unsafe abortion, Global and regional estimates of the incidence of unsafe abortion and associated mortality in 2000. World Health Organization, Geneva.

WHO, 2009. Trends in maternal mortality: 1990-2008. Estimates developed by WHO, UNICEF, UNFPA and The World Bank. World Health Organization, Geneva.

Winikoff B. Acceptability of medical abortion in early pregnancy. Fam Plan Persp 1995;27:142-148.

von Hertzen H, Honkanen H, Piaggio G, et al. WHO multinational study of three misoprostol regimens after mifepristone for early medical abortion: 1. Efficacy. *British Medical Journal* 2003.

[von Hertzen H](http://www.ncbi.nlm.nih.gov/pubmed?term="von Hertzen H"%5BAuthor%5D), [Huong NT](http://www.ncbi.nlm.nih.gov/pubmed?term="Huong NT"%5BAuthor%5D), [Piaggio G](http://www.ncbi.nlm.nih.gov/pubmed?term="Piaggio G"%5BAuthor%5D), [Bayalag M](http://www.ncbi.nlm.nih.gov/pubmed?term="Bayalag M"%5BAuthor%5D), [Cabezas E](http://www.ncbi.nlm.nih.gov/pubmed?term="Cabezas E"%5BAuthor%5D), [Fang AH](http://www.ncbi.nlm.nih.gov/pubmed?term="Fang AH"%5BAuthor%5D), [Gemzell-Danielsson K](http://www.ncbi.nlm.nih.gov/pubmed?term="Gemzell-Danielsson K"%5BAuthor%5D), [Hinh ND](http://www.ncbi.nlm.nih.gov/pubmed?term="Hinh ND"%5BAuthor%5D), [Mittal S](http://www.ncbi.nlm.nih.gov/pubmed?term="Mittal S"%5BAuthor%5D), [Ng EH](http://www.ncbi.nlm.nih.gov/pubmed?term="Ng EH"%5BAuthor%5D), [Chaturachinda K](http://www.ncbi.nlm.nih.gov/pubmed?term="Chaturachinda K"%5BAuthor%5D), [Pinter B](http://www.ncbi.nlm.nih.gov/pubmed?term="Pinter B"%5BAuthor%5D), [Puscasiu L](http://www.ncbi.nlm.nih.gov/pubmed?term="Puscasiu L"%5BAuthor%5D), [Savardekar L](http://www.ncbi.nlm.nih.gov/pubmed?term="Savardekar L"%5BAuthor%5D), [Shenoy S](http://www.ncbi.nlm.nih.gov/pubmed?term="Shenoy S"%5BAuthor%5D), [Khomassuridge A](http://www.ncbi.nlm.nih.gov/pubmed?term="Khomassuridge A"%5BAuthor%5D), [Tuyet HT](http://www.ncbi.nlm.nih.gov/pubmed?term="Tuyet HT"%5BAuthor%5D), [Velasco A](http://www.ncbi.nlm.nih.gov/pubmed?term="Velasco A"%5BAuthor%5D), [Peregoudov A](http://www.ncbi.nlm.nih.gov/pubmed?term="Peregoudov A"%5BAuthor%5D)Misoprostol dose and route after mifepristone for early medical abortion: a randomised controlled noninferiority trial.; [WHO Research Group on Postovulatory Methods of Fertility Regulation](http://www.ncbi.nlm.nih.gov/pubmed?term="WHO Research Group on Postovulatory Methods of Fertility Regulation"%5BCorporate Author%5D). BJOG 2010;117(10):1186-1196.

[Yarnall J](http://www.ncbi.nlm.nih.gov.www.bibproxy.du.se/pubmed?term="Yarnall J"%5BAuthor%5D&itool=EntrezSystem2.PEntrez.Pubmed.Pubmed_ResultsPanel.Pubmed_RVAbstract), [Swica Y](http://www.ncbi.nlm.nih.gov.www.bibproxy.du.se/pubmed?term="Swica Y"%5BAuthor%5D&itool=EntrezSystem2.PEntrez.Pubmed.Pubmed_ResultsPanel.Pubmed_RVAbstract), [Winikoff B](http://www.ncbi.nlm.nih.gov.www.bibproxy.du.se/pubmed?term="Winikoff B"%5BAuthor%5D&itool=EntrezSystem2.PEntrez.Pubmed.Pubmed_ResultsPanel.Pubmed_RVAbstract). Non-physician clinicians can safely provide first trimester medical abortion. [*Reprod Health Matters*.](javascript:AL_get(this, 'jour', 'Reprod Health Matters.');) 2009 May;17(33):61-9

1. [↑](#endnote-ref-2)
