# Supplementary material for: Acceptability of Home-Assessment Post Medical Abortion and Medical Abortion in a Low-Resource Setting in Rajasthan, India. Secondary Outcome Analysis of a Non-Inferiority Randomized Controlled Trial
Source: PLoS One. 2015 Sep 1;10(9):e0133354. doi: 10.1371/journal.pone.0133354 (PMC4556554; doi:10.1371/journal.pone.0133354)
Supplement: S1 Table — The table reflects factors associated with future preference of clinic FU or according to doctor’s advice among women in the home-assessment group. (DOC) [file pone.0133354.s003.doc]

**Supplementary Information – S1**

S1 Table. Factors among home-assessment group influencing the future preference of a clinic FU or according to doctor’s advice for method of follow-up in the event an abortion (n=349).

|  | **OR** | **CI** | **AOR** | **CI** |
| --- | --- | --- | --- | --- |
| **Advised return to clinic at the time of follow-up** |  |  |  |  |
| No | 1 |  |  |  |
| Yes | **2.4*** | 1.21-4.59 | **3.6*** | 1.58-8.33 |
| **Woman thinks her abortion is complete at follow-up** |  |  |  |  |
| Yes | 1 |  |  |  |
| No | **4.0*** | 1.60-10.19 |  |  |
| **Woman reported any remaining symptoms at follow-up** |  |  |  |  |
| No | 1 |  |  |  |
| Yes | 1.2 | 0.64-2.27 |  |  |
| **Woman still suffered from severe stomach pain** |  |  |  |  |
| No | 1 |  |  |  |
| Yes | **3.0*** | 1.13-8.14 |  |  |
| **The woman used the Pictorial instruction sheet** |  |  |  |  |
| Yes | 1 |  |  |  |
| No | **3.5*** | 1.95-6.34 | **6.1*** | 2.41-15.39 |
| **The woman did the LSUP-test by herself before follow-up** |  |  |  |  |
| Yes | 1 |  |  |  |
| No | 1.7 | 0.95-3.21 |  |  |
| **The woman found the LSUP-test easy to do** |  |  |  |  |
| Yes | 1 |  |  |  |
| No | 1.3 | 0.14-11.61 |  |  |
| **The result of the LSUP-test at follow-up** |  |  |  |  |
| Negative | 1 |  |  |  |
| Positive/Not sure | **5.0*** | 1.89-13.07 |  |  |
| **Abortion Outcome** |  |  |  |  |
| Successful | 1 |  |  |  |
| Not successful | **3.1*** | 1.08-9.16 |  |  |

Odds Ratio (OR) and Adjusted Odds Ratio (AOR) followed by a * are significantly associated to have an effect on the abortion experience. The variables that were significant in the bivariate logistic regression were further analysed in the multivariate logistic regression by using backward selection. The table shows AOR for the variables that were significant in the multivariate analysis. Due to the few women being dissatisfied several of the significant OR are not significant in the AOR, however the significant OR should not be undermined.
