# Supplementary material for: Acceptability of Home-Assessment Post Medical Abortion and Medical Abortion in a Low-Resource Setting in Rajasthan, India. Secondary Outcome Analysis of a Non-Inferiority Randomized Controlled Trial
Source: PLoS One. 2015 Sep 1;10(9):e0133354. doi: 10.1371/journal.pone.0133354 (PMC4556554; doi:10.1371/journal.pone.0133354)
Supplement: S2 Table — Results are presented as the Odds Ratio (OR) or adjusted OR of being dissatisfied. (DOCX) [file pone.0133354.s004.docx]

**Supplementary Information – S2**

**S2 Table. Characteristics of women and abortion experience in relation to satisfaction with the abortion procedure, presented as the OR/AOR of being dissatisfied.**

|  | **OR** | **95% CI** | **AOR** | **95% CI** |
| --- | --- | --- | --- | --- |
| **Group Allocation** |  |  |  |  |
| - Home-based assessment group | 1 |  |  |  |
| - Clinic follow-up group | 1.1 | 0.50-2.51 | 1.0 | 0.34-3.01 |
| **Residence** |  |  |  |  |
| - Rural | 1 |  |  |  |
| - Urban | 2.1 | 0.92-4.76 |  |  |
| **Caste/Social group** |  |  |  |  |
| - SC/ST | 1 |  |  |  |
| - Other | 1.4 | 0.64-3.27 |  |  |
| **Occupation** |  |  |  |  |
| - Paid employment | 1 |  |  |  |
| - Unpaid employment | 1.6 | 0.46-5.34 |  |  |
| **Formal education** |  |  |  |  |
| Yes | 1 |  |  |  |
| No | 1.6 | 0.67-3.63 |  |  |
| **Had someone accompanying to the clinic on day 1** |  |  |  |  |
| Somebody^a^ | 1 |  |  |  |
| Nobody | 1.8 | 0.80-4.15 |  |  |
| **Gestational age in weeks** | **1.0** | **0.74-1.44** |  |  |
| **Previous elective abortion(s)** |  |  |  |  |
| None | 1 |  |  |  |
| One or more | 2.0 | 0.89-4.57 |  |  |
| **Time spent travelling to the clinic (increased OR per hour spent)** | **1.6*** | **1.09-2.28** | **1.7*** | **1.00-2.84** |
| **Time spent in the clinic (increased OR per hour spent)** | **1.0** | **0.91-1.17** |  |  |
| **Interim visit for any reason** |  |  |  |  |
| No | 1 |  |  |  |
| Yes | **5.4*** | 2.33-12.27 |  |  |
| **Side-effects^b^ during miso-use at clinic or in retrospect at follow-up** |  |  |  |  |
| No | 1 |  |  |  |
| Yes | **3.5*** | 1.55-8.02 | **2.9*** | 1.03-8.30 |
| **Reason for additional contact** |  |  |  |  |
| **Other reasons** | **1** |  |  |  |
| **Related to complication/side-effect^c^** | **12.5*** | **5.29-29.75** | **4.7*** | **1.73-12.91** |
| **Additional Surgical/medical intervention to complete abortion** |  |  |  |  |
| No | 1 |  |  |  |
| Yes | **26.1*** | 10.56-64.70 |  |  |
| **Reported symptoms^d^ at follow-up** |  |  |  |  |
| None/ Not abortion related | 1 |  |  |  |
| Abortion related | **6.8*** | 2.21-20.98 | **4.7*** | 1.67-13.10 |
| **Outcome of abortion** |  |  |  |  |
| Complete abortion | 1 |  |  |  |
| On-going pregnancy/ incomplete abortion | **27.4*** | 11.03-68.21 | **18.0*** | 5.67-56.80 |

Odds Ratio (OR) and Adjusted Odds Ratio (AOR) followed by a * are significantly associated to have an effect on the abortion experience. The variables that were significant in the bivariate logistic regression were further analysed in the multivariate logistic regression by using backward selection. The table shows AOR for the group allocation of the women and variables that were significant in the multivariate analysis.

^a^ Somebody include husband, health staff, maternal family member, in-law family member, neighbour, friend, previous patient.

^b^ Side effects include nausea, vomiting, diarrhoea, sever pain, fever, chills, excessive bleeding, or giddiness

^c^ Complications/ side effects include excessive bleeding, pain, fever, giddiness, weakness, and little bleeding.

^d^ Symptoms include weakness, body ache, excessive bleeding, stomach pain, not feeling well and continuing symptoms of pregnancy. Only symptoms remaining at follow-up.
